# Yield Performance, Combining Ability and Stability of Early- to Medium-Maturing Doubled-Haploid Maize Lines in Eastern Africa

**DOI:** 10.1111/pbr.70072

**Published:** 2026-03-10

**Authors:** Wilson Mwangangi, Manje Gowda, Kulai Amadu Manigben, Felister Mbute Nzuve, Lydia Nanjala Wamalwa, Juan Burgueno, Vijay Chaikam, Yoseph Beyene

**Affiliations:** 1 International Maize and Wheat Improvement Center (CIMMYT), World Agroforestry Centre (ICRAF), United Nations Avenue, Gigiri, Nairobi, Kenya; 2 Department of Plant Science and Crop Protection, University of Nairobi, Nairobi, Kenya; 3 West Africa Centre for Crop Improvement (WACCI), University of Ghana, Accra, Ghana; 4 CSIR-Savanna Agricultural Research Institute, Nyankpala, Ghana; 5 International Maize and Wheat Improvement Centre (CIMMYT), Mexico City, Mexico

**Keywords:** general combining ability, GGE biplot, grain yield, line × tester, maize, stability

## Abstract

Maize is a staple food crop in sub-Saharan Africa, where increasing productivity is essential for food security. This study assessed testcross performance and combining ability of early- to medium-maturity doubled haploid (DH) maize lines adapted to eastern Africa. A total of 732 testcross hybrids derived from 244 DH lines crossed with six testers, along with six commercial checks, were evaluated across eight locations. Agronomic and disease-related traits were recorded and analysed using mixed models, line × tester analysis and stability tools (AMMI and GGE biplots). Significant genetic variation (*p* ≤ 0.05) was detected among genotypes, environments and their interactions. Additive genetic effects were predominant, as indicated by high Baker’s ratios (> 0.70) and narrow-sense heritability estimates (0.71–0.95), highlighting the importance of general combining ability (GCA) in hybrid prediction. Several lines, including CKDHL141370, CKDHL142389 and CKDHL143580, exhibited favourable GCA effects for grain yield and earliness, while also contributing to resistance against turcicum leaf blight and grey leaf spot. The top-performing hybrids, such as (CKDHL120312/CML312)//CKDHL142124, CKDHL143094 and CKDHL142908, yielded 7.4–8.2 t ha^−1^, representing a 29%–79% advantage over commercial checks. GGE biplot analysis explained 71.27% of the variation and identified several hybrids combined high yield with stability across environments. Overall, these results demonstrate that integrating line × tester and GGE biplot approaches is highly effective for identifying superior parental lines and predicting their hybrid performance. The identified superior hybrids warrant further validation in on-farm trials for potential commercialization to enhance maize productivity and resilience in sub-Saharan Africa.

## 1 | Introduction

Maize (*Zea mays* L., 2n = 2x = 20) is a staple crop in sub-Saharan Africa (SSA), where over 80% of the population relies on it for food, income and livelihoods (Pardey et al. 2016; Prasanna et al. 2020, 2022; Kibe et al. 2020). Although Africa accounts for ~20.9% of the global maize production area, it contributes only ~7.4% of total global output. This low productivity is driven by a complex set of factors, including biotic and abiotic stresses, limited input use and sub-optimal crop management practices resulting from weak extension systems. Enhancing maize yields in SSA therefore requires a deeper understanding of tropical maize breeding and the deployment of stress-resilient, high-yielding varieties. Between 2020 and 2022, global maize production declined by ~4% (~44 million metric tons) due to climate variability and recurrent drought (FAO 2023), whereas demand continues to rise and is projected to surpass 700 million metric tons annually (Erenstein et al. 2022; Ranum et al. 2014).

In East Africa, maize is central to food security. In Kenya, for example, it occupies ~2.1 million hectares—nearly 40% of harvested cropland (FAO 2023). Despite this importance, national maize yields average only 1.5–2.2 t ha^−1^, resulting in an annual deficit of ~1 million metric tons (Masese et al. 2022), often met by overpriced imports valued at US$ 143 million in 2023. Maize contributes ~28% to agricultural GDP and supports three-quarters of rural households, yet production remains constrained by limited arable land, erratic rainfall and frequent pest and disease outbreaks (Prasanna et al. 2021). With Kenya’s population projected to exceed 91 million by 2050 (Statista 2024), the urgency to boost maize productivity under climate uncertainty is increasing.

Hybrid breeding remains one of the most effective strategies to consider to enhance maize yields in such environments. The success of hybrid development depends on identifying superior parental lines with desirable genetic potential, measured through combining ability. General combining ability (GCA) reflects additive gene effects and indicates the average performance of a line across hybrid combinations, whereas specific combining ability (SCA) reflects non-additive gene effects such as dominance and epistasis and reveals the potential of parental combinations (Manigben et al. 2024; Kamweru, Beyene, Anani, et al. 2023; Kamweru, Beyene, Bruce, et al. 2023). Both GCA and SCA are therefore critical for selecting parents and predicting hybrid performance.

In tropical maize, previous studies have reported the contributions of additive and non-additive gene actions to grain yield and other traits (Kamweru, Beyene, Anani, et al. 2023; Nzuve et al. 2014; Mary 2016; Manigben et al. 2024; Mebratu et al. 2024). These findings underscore the importance of routinely assessing new and existing parental lines for combining ability, especially in stress-prone environments. Genetic diversity among parental lines, often assessed through morphological and molecular markers, forms the basis for heterosis exploitation and hybrid prediction (Crossa et al. 2017). Understanding the relationship between genetic distance, heterosis and combining ability can further refine parental selection strategies.

The line × tester (L × T) mating design (Kempthorne 1957) is widely used in maize breeding for simultaneously evaluating the GCA of multiple parental lines and the SCA of their testcross hybrids. Its efficiency in identifying elite parents and superior hybrids has been demonstrated across many tropical breeding programmes (Betrán et al. 2003; Makumbi et al. 2011; Badu-Apraku et al. 2015; Trachsel et al. 2016; Beyene et al. 2017; Ertiro et al. 2017; Muthoni and Shimelis 2020; Osuman et al. 2022; Sang et al. 2022; Matongera et al. 2023). Beyond combining ability analysis, breeders also examine phenotypic and genotypic variances, heritability and trait correlations to guide selection (Nzuve et al. 2014). For grain yield, a complex trait with low to moderate heritability (Musvosvi et al. 2018), the L × T design provides valuable insights into parental contributions and hybrid potential (Abrha et al. 2013).

Multi-environment trials (METs) are fundamental in maize breeding as they capture genotype × environment interactions (GEIs) that determine hybrid performance across diverse environments. GEIs complicate selection, advancement and recommendation, particularly in East Africa, where production environments are highly variable (Prasanna et al. 2021). To address this challenge, breeders focus on adaptability—a genotype’s ability to exploit favourable conditions—and stability—its consistency under diverse and changing environments. Hybrids that combine both traits are most valuable for broad deployment.

Among the methods developed to analyse GEIs, the GGE biplot (genotype main effects + genotype × environment interaction) has become widely adopted (Yan et al. 2000). This approach graphically integrates genotype performance and interaction patterns, enabling the identification of superior hybrids, classification of test environments and delineation of mega-environments. Its visual interpretability and statistical power make it particularly effective for guiding regional varieties recommendations. The utility of GGE biplots has been demonstrated across maize studies. Berhanu et al. (2024), Gonçalves et al. (2025), Lima et al. (2023) and Oliveira et al. (2019) identified stable, high-yielding hybrids, while Crevelari et al. (2023) applied it to silage maize and Santos et al. (2017) to popcorn maize, underscoring its versatility. Here, we applied GGE biplot analysis to evaluate maize hybrids across contrasting environments in Kenya, representing the target production environments of the East African mid-altitude region, with the objective of identifying high-yielding, stable and widely adaptable genotypes for regional recommendation.

Genetic improvement of maize hybrids can be achieved either by enhancing the performance of parental lines or by exploiting heterosis in their combinations. In stress-prone tropical environments such as Eastern Africa, both strategies are vital for sustainable productivity gains. Evaluating large sets of early- to medium-maturity maize lines for combining ability provides an opportunity to identify elite parents and hybrids adapted to diverse agro-ecological conditions. This study aimed to (i) evaluate the phenotypic performance of testcross hybrids, (ii) estimate GCA and SCA variances for grain yield and key agronomic traits, (iii) assess trait correlations relevant to hybrid performance and (iv) apply GGE biplot analysis to identify high-yielding, stable and widely adaptable genotypes across multiple environments. The results will inform parental selection, hybrid prediction and the development of high-yielding maize hybrids adapted to Eastern Africa.

## 2 | Materials and Methods

### 2.1 | Plant Materials, Field Experiments, Data Collection and Phenotypic Data Analysis

The doubled haploid (DH) lines used in this study were derived from the International Maize and Wheat Improvement Center’s (CIMMYT) East Africa mid-altitude medium maturity breeding programme. These DH lines were developed at CIMMYT’s DH facility at Kiboko in Kenya from the biparental crosses between selected multiple stress tolerant elite lines. The 244 selected DH lines were represented by heterotic group A (*n* = 162) and heterotic group B (*n* = 82). An incomplete line-by-tester design (Kempthorne 1957) was used to generate 732 testcrosses by crossing 244 lines to six single-cross testers (Table S1). Pollen from each line was used to manually pollinate the single-cross testers. The testcrosses were formed during the short rainy season (October–March) in the Kiboko maize research station in Kenya. The parental lines were selected through rigorous phenotypic evaluations in the breeding nurseries and screened for foliar diseases. The testers used in the study are good combiners and are being used as female parents to make a three-way hybrid adapted to mid-altitude environments (Beyene et al. 2017; Ertiro et al. 2017; Gowda et al. 2026).

### 2.2 | Experimental Design and Management

All 732 testcross hybrids and six commercial checks were planted in an alpha lattice experimental design with two replications evaluated in eight locations under optimum conditions (Table 1). To minimize field heterogeneity and reduce error variance, the experiment was subdivided into 12 smaller trials. The 732 experimental hybrids and 6 commercial checks were distributed across these trials, each containing 66 entries (60 experimental hybrids and 6 checks) evaluated in two replications. At all sites, the entries were hand-planted in two-row plots, 5 m long each, with 0.75-m spacing between rows and 0.25 m between hills. Two seeds were initially planted per hill and later thinned to one plant per hill at 3 weeks after seedling emergence to adjust the final plant population to 53,333 plants/ha. Basal fertilizer application was performed at planting using di-ammonium phosphate (DAP) fertilizer at the rate of 60-kg N and 60-kg P_2_O_5_ per hectare. Six weeks after emergence, all experiments were top-dressed with nitrogen fertilizer at the rate of 60-kg nitrogen per hectare. All the experiments were kept weeds-free by manual weeding and herbicide control.

Data were recorded on grain yield (GY), anthesis date (AD), silking date (SD), ears per plant (EPP), plant height (PH), ear rot (ER), moisture content (MOI), turcicum leaf blight (TLB) and grey leaf spot (GLS) disease severity. GY was measured in tons per hectare adjusted to a grain moisture content (MOI) of 12.5% by using a portable moisture tester (DICKEY-john). AD was recorded by counting the total number of days from planting to when 50% of plants in a plot started shedding pollen in the primary tassel axis; PH was measured in centimetres from the base of the plant to the first branch of the tassel; EPP per plot was obtained by dividing the total number of ears harvested from a plot by the total number of plants at harvest. In the optimum experiments, ears harvested from each plot were weighed and subsamples were shelled to determine representative grain moisture. GY was estimated to have a shelling percentage of 80% and adjusted to 12.5% moisture content. Testcross hybrids were evaluated for GLS and TLB under natural disease pressure at the hotspot areas in western Kenya (Suresh et al. 2025). GLS severity was recorded at mid-silking and hard dough stages, while TLB severity was assessed at the hard dough stage. Disease severity for both traits was scored plot-wise using a standardized 1–9 ordinal scale, where 1 indicated highly resistant plants with no visible symptoms and 9 indicated highly susceptible plants with extensive necrosis or plant death, reflecting increasing lesion abundance and leaf area damage.

### 2.3 | Data Analysis

Phenotypic data analyses for each and across locations were carried out using Multi-Environment Trait Analysis software in the R environment (META-R) (Alvarado et al. 2020). Best linear unbiased predictions (BLUPs) and best linear unbiased estimates (BLUEs) for each genotype were generated for each and across locations (Tables S2 and S3). Analysis of variance was determined for GY and other yield-related traits by restricted maximum likelihood method using META-R. The linear mixed model used for across locations analyses was

$$\text{Trait}=\text{mean}+\text{environment}+\text{replication}\;\left(\text{environment}\right)+\text{block}\;\left(\text{replication}\left(\text{environment}\right)\right)+\text{Genotype}+\text{Genotype}*\text{environment}+\text{residual}\;(\text{Model}\;1)$$

Environment and replication nested in environment were considered as fixed effects while the other effects in the model were random effects with normal distribution, zero mean and pairwise independent. Broad sense heritability was computed as the ratio of phenotypic variance to the genotypic variance based on the entry means (Hallauer et al. 2010). The correlation between traits was calculated using the *cor* function in R (R Core Team 2023). Correlation heatmap and distribution of phenotypic values of the traits were generated using the *gg-plot2* R package.

To conduct line-by-tester data analyses across environments, checks were excluded. The total variance of the testcross hybrid values was partitioned into the GCA variance due to line and tester as well as SCA variance due to line × tester (Liu et al. 2021; Trachsel et al. 2016). Data were analysed using the AGD-R version 4 software (Rodríguez et al. 2015). Analyses were performed for within and across locations using joint linear mixed models such as

Trait=mean+environment+replication(environment)+block(replication(environment))+Line+Tester+Line*Tester+Line*Environment+Tester*Environment+Line*Tester*Environment+error(Model2)

The effects of environment and replication within environment were considered fixed, while line, tester, line × tester and block within environment within replication were considered random. The restricted maximum likelihood procedure was used to estimate all variance components (Rodríguez et al. 2015). Test of significance of the variance component estimates and model comparison were performed using the likelihood ratio test (Giampaoli and Singer 2009; Stram and Lee 1994).

### 2.4 | Estimation of GCA and SCA Effects

The GCA effects for lines and testers, and the SCA effects for line × tester interactions, were estimated following the procedure of Singh and Chaudhary (1985). The significance of GCA and SCA effects at the 5% probability level was tested by dividing each GCA or SCA estimate by its corresponding standard error to obtain the *t*-value, which was then compared with the tabulated *t*-value at the appropriate error degrees of freedom (Singh and Chaudhary 1985). The relative importance of GCA and SCA was assessed using Baker’s ratio, calculated from the variance components of GCA (lines and testers) and SCA (testcross hybrids). A Baker’s ratio value approaching unity indicates greater predictability of hybrid performance based solely on GCA effects (Baker 1978). The proportional contribution of total genetic variance due to lines, testers and the interaction between lines and testers was computed (Singh and Chaudhary 1985).

The best yielding 42 testcross hybrids and six commercial checks and GY data (BLUE values) from four locations (Embu, KYUC, Kakamega, Mbeere) in Kenya were subjected to GGE biplot analysis (Yan 2001) to decompose the G × E interactions (Table S4). The biplot was generated using the first two principal components (PCs) according to the column metric preserving (‘GH’) GGE model for singular value partitioning. The data were centred using the ‘tester-centred G + GE’ approach without any data scaling. The GGE biplot analyses were done using GEA-genotype × environment analysis with R for Windows version 4.1 (Pacheco et al. 2018).

## 3 | Results

### 3.1 | Phenotypic Variation and Trait Distributions

**FIGURE 1 |:**
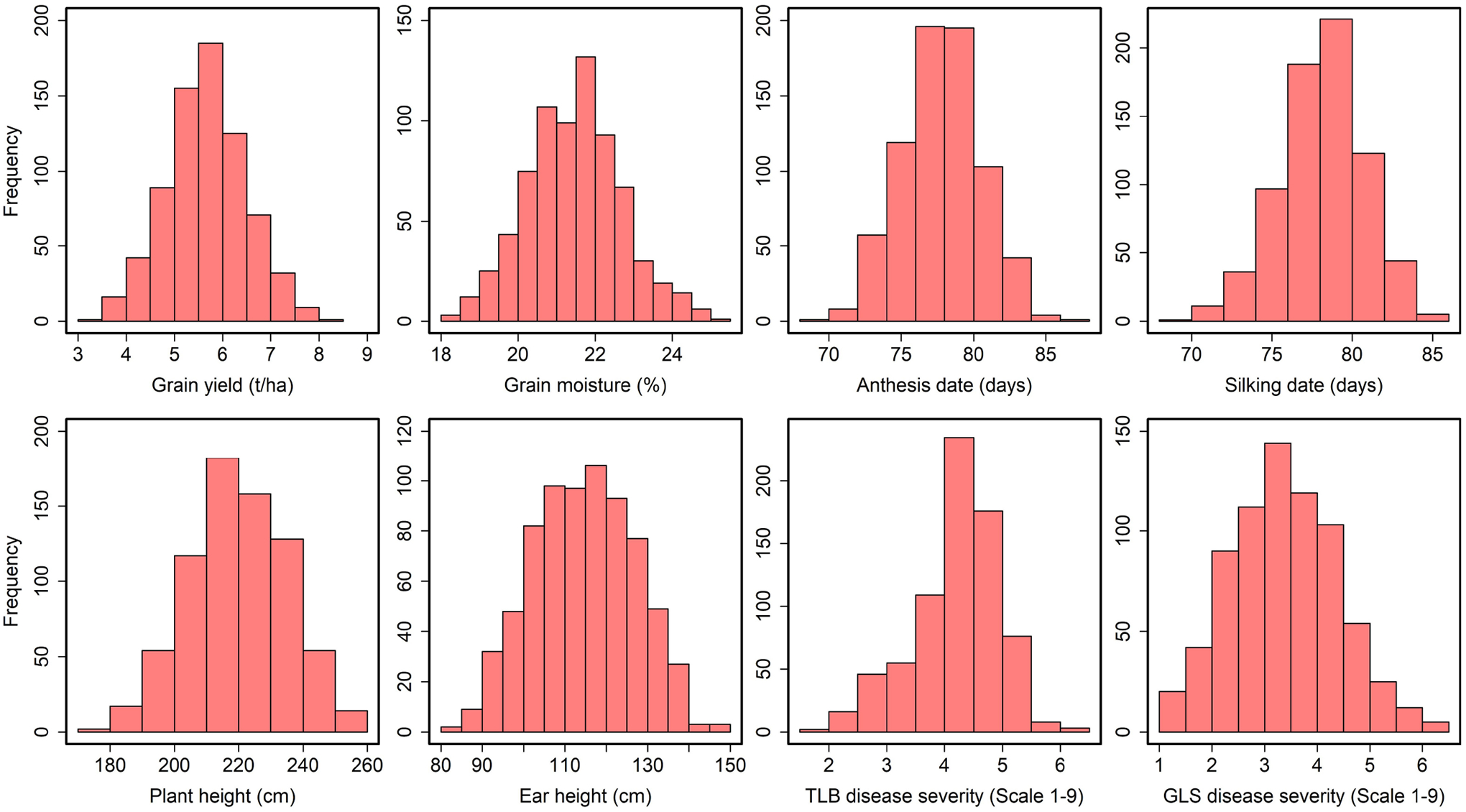
Frequency distribution of 732 testcross hybrids for GY and other agronomic traits evaluated in eight different locations in Kenya.

Significant genetic variation was observed among the testcross hybrids for GY and other agronomic traits across environments (Table 2). The phenotypic distributions for GY and related traits were continuous and approximately normal, indicating quantitative inheritance (Figure 1). Selected best 25 testcross hybrids outperformed commercial checks for GY and disease resistance (Table 3). GY of the top 25 experimental hybrids ranged from 7.17 to 8.19 t ha^−1^, markedly higher than the six commercial checks, which yielded between 4.57 and 6.34 t ha^−1^ (mean yield of 5.39 t ha^−1^). The highest yielding hybrid—(CKDHL120312/CML312) X CKDHL142124—produced 8.19 t ha^−1^, representing a yield advantage of 29%–79% over the checks. AD and SD ranged from 74–83 days, with a mean of 78 days, indicating moderate earliness compared with checks such as Duma43 (69.5 days). PH among the best 25 hybrids varied from 206 to 252 cm, with ear placement (EH) between 102 and 139 cm, generally taller than checks but within an acceptable range for lodging tolerance (Table 3).

Disease severity scores showed that most best yielding hybrids had lower severity for TLB (1.72–3.03) and GLS (1.38–3.32) compared with checks (TLB: 3.85–4.58; GLS: 3.18–4.03). Similarly, ER incidence was much reduced in the top hybrids (0.85%–11.97%) relative to checks (7.74%–12.81%). For grain moisture (MOI), testcrosses averaged 22%, slightly higher than checks (19%–22%), but still within acceptable harvest moisture range (Table 3). Husk cover (HC) varied widely, with most hybrids showing favourable tight HC (1%–13%), whereas some checks exhibited higher values (> 10%). Overall, the superior performance of the best testcross hybrids for yield and disease resistance, combined with acceptable agronomic traits, highlights their potential for advancement to multilocation trials and possible release as climate-resilient, high-yielding maize hybrids.

**FIGURE 2 |:**
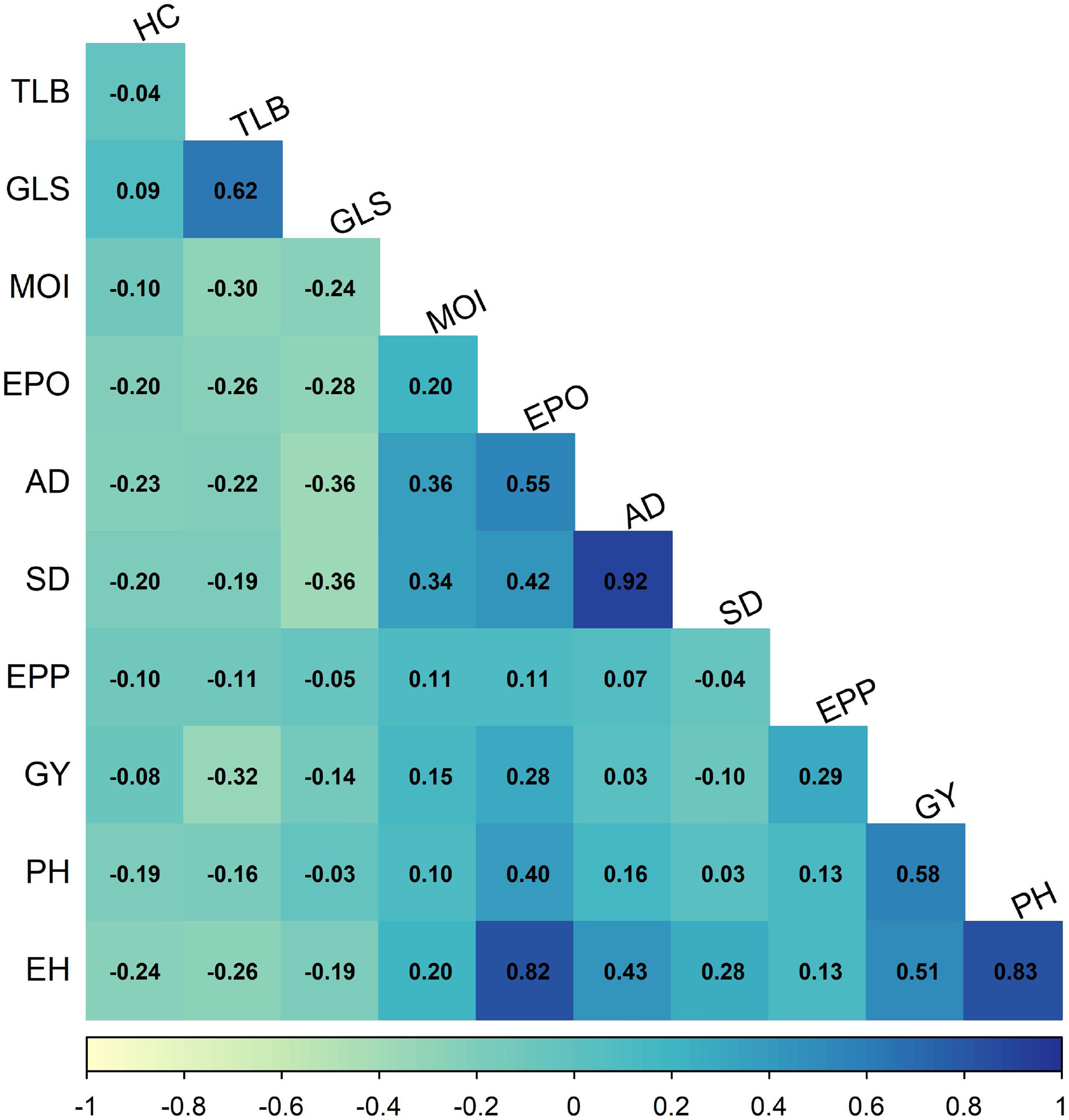
Pearson’s correlation between grain yield and other traits evaluated in multiple environments. The correlation level is colour-coded according to the colour key indicated on the scale. Correlations with > 0.15 were significant at 0.05 (*p*) level. AD, days to 50% anthesis; EH, ear height; EPO, ear position; EPP, ears per plant; GLS, grey leaf spot; GY, grain yield; MOI, moisture content; PH, plant height; SD, silking date; TLB, turcicum leaf blight.

Correlation analysis (Figure 2) revealed that grain yield was positively and significantly associated with plant height and ear height, while negatively correlated with moisture content at harvest. Weak but significant negative correlations were observed between yield and disease resistance traits, suggesting that higher-yielding hybrids also tended to express moderate resistance to TLB and GLS. Days to anthesis and silking were highly correlated (*r* > 0.90, *p* < 0.01), and both showed weak negative correlations with grain yield. Flowering traits and HC were significant and negatively correlated with TLB and GLS disease severity.

### 3.2 | Variance Components and Genetic Control

Partitioning of variance components for GY and associated traits under optimal conditions revealed significant contributions from both lines and testers (Table 4). Line variance (GCA of lines) was highly significant for all traits, ranging from 0.24 for GY to 112.98 for PH, indicating strong genetic variability among the parental lines. Tester variance was also significant but generally lower than line variance, with the highest values observed for PH (11.46) and EH (8.23), while negligible variance was detected for GY. The line × tester interaction variances were significant but small for most traits, suggesting limited but non-negligible non-additive genetic effects.

Genotypic variance was consistently higher than environmental variance across all traits, reflecting strong genetic control. Additive variance was the predominant component, with particularly high estimates for GY (1.10), PH (475.75) and EH (373.23), whereas dominance variance was near zero for most traits, underscoring the predominance of additive gene action. Heritability estimates were high across traits. Broad-sense heritability ranged from 0.73 (MOI and TLB) to 0.97 (AD), while narrow-sense heritability closely matched, ranging from 0.71 to 0.95, indicating that much of the genetic variance was additive. Particularly high heritability was recorded for anthesis (0.95), silking (0.92), PH (0.91) and EH (0.95), suggesting reliable transmission of these traits to progeny. Baker’s ratio (ratio of GCA to total genetic variance) further supported the predominance of additive effects, with values above 0.70 for all traits, confirming the importance of GCA over SCA (Table 4).

### 3.3 | GCA Effects

**FIGURE 3 |:**
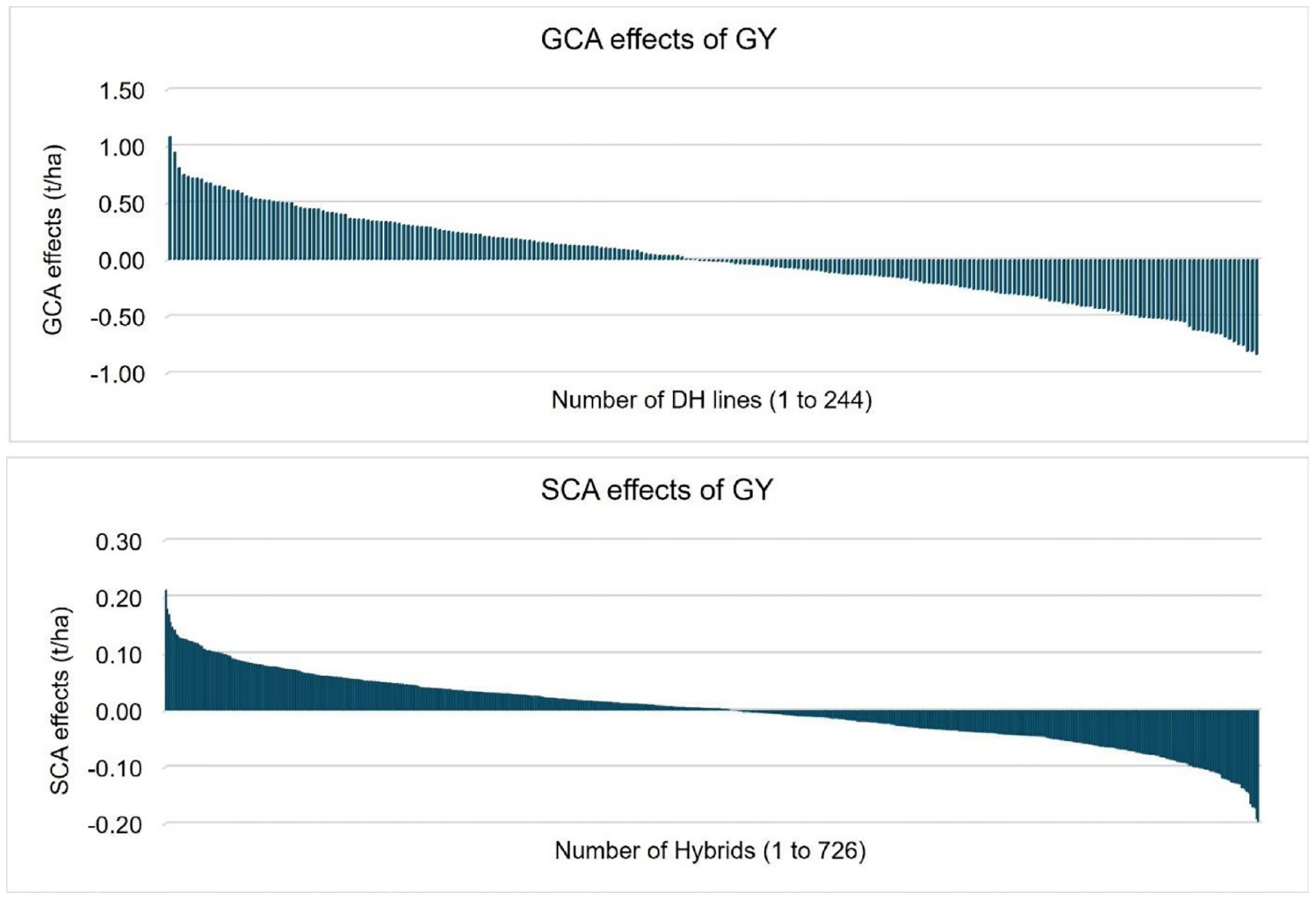
GCA and SCA estimates of 243 DH lines and 726 testcross hybrids, respectively for grain yield across locations.

The GCA analysis revealed substantial variation among the evaluated lines and testers for GY and related traits (Table 5, Figure 3). Several lines exhibited consistently positive and significant GCA effects for GY, with the best contributors being CKDHL141370 (1.09), CKDHL142389 (0.95) and CKDHL143580 (0.81). These high-yielding lines also displayed desirable negative GCA estimates for AD and SD, suggesting their potential as sources of both yield improvement and earliness. For example, CKDHL141370 combined high yield with early flowering (−2.62 AD; −1.73 SD), a trait combination particularly valuable under stress-prone environments. Other strong yield contributors such as CKDHL140502 and CKDHL142908 also enhanced PH and EH, whereas lines like CKDHL143576 and CKDHL143587 reduced plant stature, making them useful for improving lodging resistance.

Several lines also contributed positively to disease resistance. Notably, CKDHL141370, CKDHL142389 and CKDHL143580 had negative GCA estimates for TLB and GLS, indicating their value as dual-purpose donors for yield and resistance. A few lines, however, showed undesirable positive contributions to GLS, such as CKDHL140521 and CKDHL141526, highlighting the need for careful parental selection. Among the testers, most showed small GCA effects; however, tester CKDHL120312/CML312 contributed strongly to earliness (−2.57 AD; −2.99 SD), while tester CML312/CML442 showed slight positive effects for yield and GLS resistance.

Summary statistics of GCA (Table 6, Figure 3) confirmed the predominance of additive effects. Across 243 lines, GCA values for GY ranged from −0.84 to 1.09, while for PH the range was −22.01 to 21.19, indicating wide genetic variation. In contrast, SCA effects across 726 hybrids were narrow, with yield SCA effects peaking at only 0.21. The higher frequency and magnitude of positive GCA compared with SCA emphasize the importance of additive gene action, suggesting that selection based on line per se performance and GCA would be more efficient than relying on specific hybrid combinations.

### 3.4 | GGE Biplot Analysis

**FIGURE 4 |:**
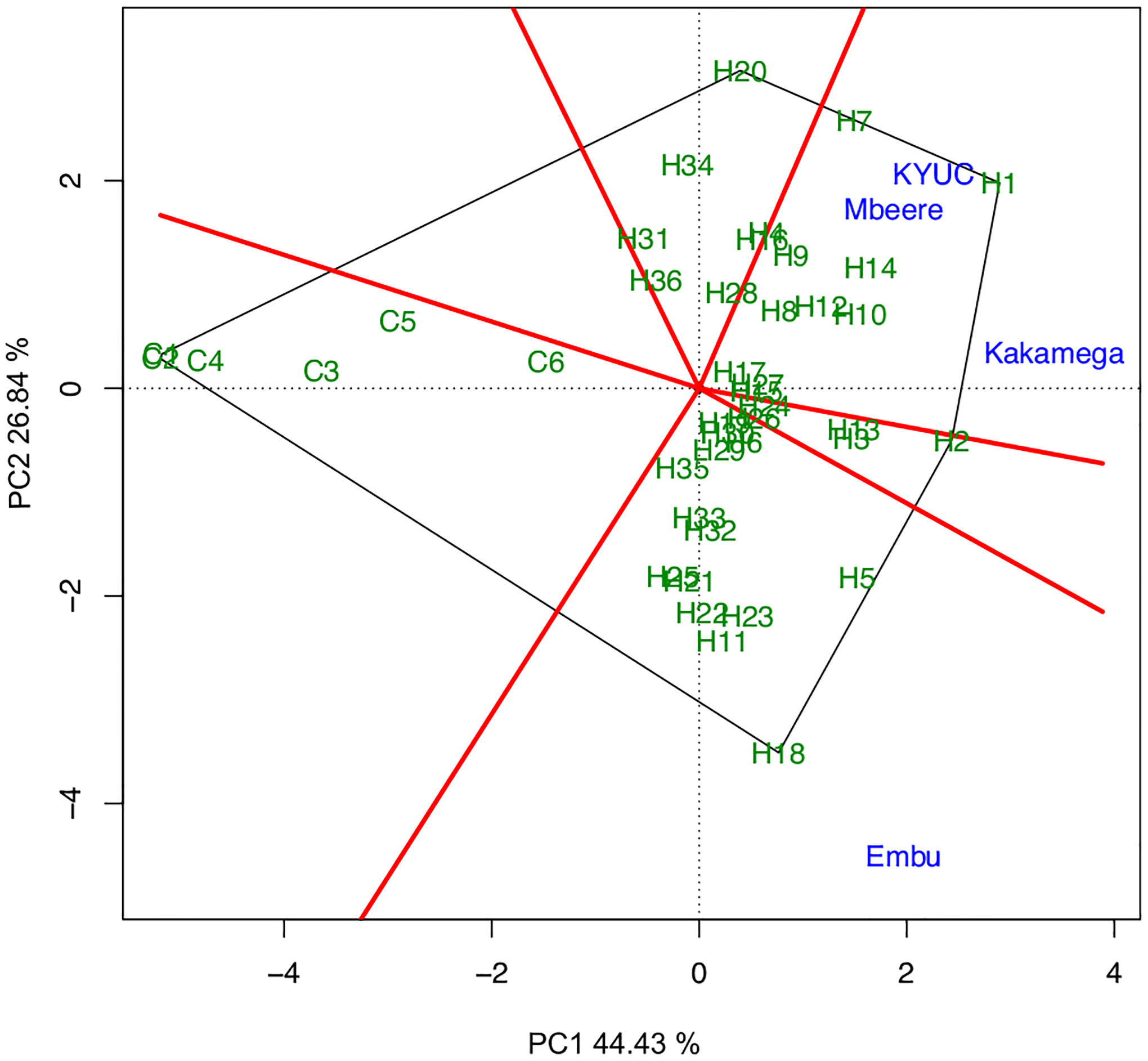
The which-won-where view of the GGE biplot show which Hybrids performed well in which locations based on genotype × environment yield data of top 42 hybrids including six commercial checks evaluated in four locations across Kenya.

The GGE biplot was constructed by plotting the scores of the first two PCs derived from the genotype × environment interaction. PC1 explained 44.43% of the total variation, while PC2 accounted for 26.84%, together explaining 71.27% of the total variation in GY of intermediate-maturity maize hybrids across four locations in Kenya. The polygon view (Figure 4) revealed five vertex hybrids (H20, H1, H2, H18 and C2) that formed the outer boundary of the biplot. The perpendicular lines from the origin partitioned the biplot into six sectors, of which the environments occupied two. Vertex hybrids within each sector were identified as the ‘winning’ genotypes, that is, those with superior yield performance in the environments falling within that sector. Hybrid H1 was the winning genotype in KYUC, Mbeere and Kakamega, while H18 was the best performer in Embu. In contrast, H2, H20 and C2 were not associated with any environment, indicating poor performance across locations. Several other hybrids (e.g., H17, H27, H15, H24, H26, H30, H19, H6, H29 and H35) clustered near the biplot origin, suggesting average performance and lower responsiveness compared with the vertex hybrids. The polygon view further delineated two mega-environments: one comprising KYUC, Mbeere and Kakamega and the other represented solely by Embu.

**FIGURE 5 |:**
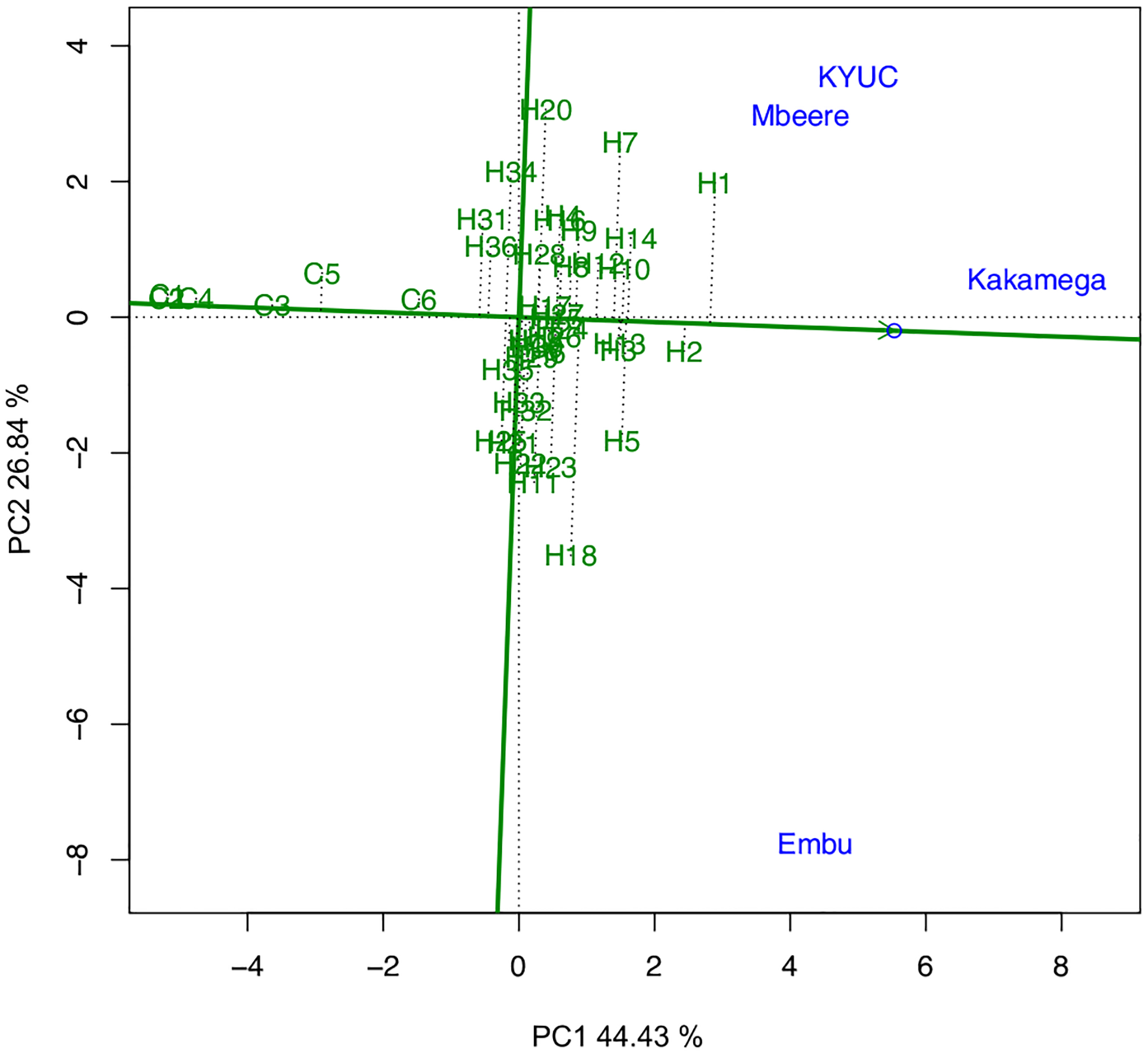
The average-environment coordination (AEC) view of the GGE biplot show the mean performance and stability of the Hybrids based on genotype × environment yield data of top 42 hybrids including six checks evaluated in four locations across Kenya.

The average-environment coordination (AEC) view (Figure 5) provided further insight into genotype performance and stability. The AEC abscissa (average environment axis) ranked genotypes by mean GY, with H1 emerging as the highest yielding hybrid, followed by H2, H13, H14, H5, H10, H3, H12 and H7, all of which had above-average means. The AEC ordinate, perpendicular to the abscissa, distinguished stable from unstable hybrids. Shorter projections onto the ordinate indicated greater stability and lower contributions to GEI. Accordingly, H2, H3, H15, H24, H17, H26, H27, H19 and H13 were identified as relatively stable and high-yielding hybrids. In contrast, check hybrids C2, C1, C3 and C6 were more stable but consistently low yielding across environments.

## 4 | Discussion

### 4.1 | Phenotypic Variation and Trait Performance

The significant phenotypic variation observed among the testcross hybrids for GY and associated traits highlights the rich genetic diversity available within the parental germplasm. The continuous and approximately normal distribution of GY and most agronomic traits confirms their quantitative inheritance, consistent with earlier reports in maize (Betrán et al. 2003; Hallauer et al. 2010). Such distributions also suggest that polygenic inheritance governs these traits, making them responsive to selection under recurrent schemes.

The superior performance of the best 25 testcross hybrids compared with commercial checks demonstrates the effectiveness of the line × tester mating design in identifying superior heterotic combinations. The top-yielding hybrid—(CKDHL120312/CML312) X CKDHL142124—yielded 8.19 t ha^−1^, representing a substantial yield advantage of up to 79% over the commercial checks. Yield superiority of this magnitude is critical for closing the yield gap in SSA, where the average maize yield remains below 2.5 t ha^−1^ (Prasanna et al. 2021). Similar yield advantages of experimental hybrids over commercial checks have been reported in CIMMYT breeding programmes targeting eastern and southern Africa (Beyene et al. 2013, 2017, 2019, 2021), underscoring the success of modern breeding approaches in generating climate-resilient, high-yielding maize.

Importantly, the high-yielding hybrids also exhibited desirable disease resistance and agronomic traits. Most top hybrids showed reduced severity for TLB and GLS, with scores consistently lower than those of commercial checks. This suggests that yield superiority was not achieved at the expense of disease resistance, which is critical for enhancing adoption and farmer confidence. The reduced incidence of ER among experimental hybrids further highlights their potential to contribute to grain quality and food safety, given the strong link between ER and mycotoxin contamination (Munkvold 2003; Shu et al. 2017; Ndlovu et al. 2022, 2024). Although grain moisture content in the experimental hybrids was slightly higher than checks, the values remained within acceptable harvest limits and the presence of tight HC in most hybrids further mitigates risks of post-harvest losses.

### 4.2 | Trait Relationships and Selection Implications

Correlation analysis revealed positive associations of GY with plant and ear height, consistent with findings of previous studies (Bänziger 2000; Kibe et al. 2020; Amadu et al. 2025). Although taller plants may enhance biomass and assimilate partitioning to the grain, excessively tall hybrids can be prone to lodging. Therefore, identification of high-yielding hybrids with moderate plant stature, such as CKDHL141370-derived combinations, is valuable for breeding lodging-tolerant yet high-yielding varieties. The negative correlation of yield with grain moisture suggests that higher yielding hybrids tend to mature later, as longer grain filling often results in higher moisture at harvest. Although this relationship is expected, breeding for earliness and reduced grain moisture remains important in SSA to enable early harvest, reduce storage losses and escape terminal drought stress. The strong correlation between AD and SD (*r* > 0.90, *p* < 0.01) highlights the close genetic control of these flowering traits, while their weak negative association with yield indicates that moderate earliness can be achieved without significant yield penalties. These findings confirm earlier work by Betrán et al. (2003) and Beyene et al. (2013), which demonstrated that synchrony of flowering and shortened anthesis–silking interval are crucial for adaptation under stress conditions.

### 4.3 | Genetic Control of Traits

Variance component analysis confirmed the predominance of genetic over environmental effects for most traits (Table 4). High line variances across traits indicate strong genetic variability among parental lines, while tester variances, though significant, were comparatively smaller. This suggests that most of the observed genetic variation originated from lines, consistent with their diverse genetic backgrounds.

The predominance of additive variance across traits, coupled with near-zero dominance variance, underscores the strong role of additive gene action in governing GY, plant stature and disease resistance. This aligns with earlier studies in maize, where additive effects were found to be the major drivers of genetic gain in recurrent selection programmes (Hallauer et al. 2010; Beyene et al. 2019; Manigben et al. 2024). The high heritability estimates for AD, SD, PH and EH indicate that these traits are reliably transmitted across generations and can be effectively improved through selection. The high Baker’s ratios (> 0.70) further confirm the pre-eminence of GCA over SCA, emphasizing the reliability of parental performance in predicting hybrid potential. This also indicates that line performance is largely predictable, and selection based on GCA would be highly effective. Overall, the results highlight that GY and other agronomic traits are largely governed by additive genetic effects, making them amenable to improvement through recurrent selection and strategic parental line development.

The implications from this study for breeding are profound. Because additive effects dominate, line per se performance and GCA estimates can serve as reliable indicators of hybrid potential, enabling breeders to accelerate selection efficiency and reduce reliance on extensive hybrid testing. This is particularly relevant for SSA, where resources for multilocation hybrid evaluation are limited.

### 4.4 | Combining Ability and Parental Contributions

The GCA analysis identified several outstanding parental lines with consistent positive contributions to GY and complementary agronomic traits (Table 5). Lines CKDHL141370, CKDHL142389 and CKDHL143580 emerged as key donors, combining high yield potential with earlier flowering through reduced AD and SD, while also conferring resistance to TLB and GLS. Such multiple-trait donors are particularly valuable for breeding programmes targeting stress-prone environments. Other lines, including CKDHL140502 and CKDHL142908, contributed positively to PH and EH, offering potential for improving plant vigor, whereas CKDHL143576 and CKDHL143587 reduced plant stature, a desirable trait for lodging tolerance. These diverse contributions highlight the opportunity to assemble complementary parents to achieve targeted trait combinations. Although testers generally showed small effects, CKDHL120312/CML312 contributed significantly to earliness and CML312/CML442 to yield and GLS resistance, suggesting that testers can add useful agronomic attributes beyond their role in heterotic group classification.

Across the panel of 243 lines, GCA values for yield ranged from −0.84 to 1.09 t ha^−1^, whereas SCA effects across 726 hybrids were much narrower, with maximum yield contributions of only 0.21 (Table 6, Figure 3). The higher magnitude and frequency of positive GCA compared with SCA emphasize that hybrid performance is largely predictable from parental performance, consistent with earlier findings (Beyene et al. 2011; Suwarno et al. 2014). Notably, 13 of the top 25 lines had high and significant GCA estimates for GY (> 0.65), reflecting the effectiveness of the breeding strategy, where several cycles of selection enriched favourable alleles. The high Baker’s ratio of GCA to SCA across all traits, including GY, further underscores the predominance of additive over non-additive gene action, in agreement with earlier studies (Worku et al. 2008; Gerhardt et al. 2019; Mutimaamba et al. 2020; Manigben et al. 2024).

From a breeding perspective, the predominance of additive variance is highly advantageous because it enables independent selection and improvement of parental lines, reduces dependence on specific hybrid combinations and accelerates genetic gain. Future breeding efforts should integrate elite lines with strong additive effects for yield with resistance to foliar diseases, stress resilience and desirable agronomic traits. In SSA, where maize is a staple food and income source, this approach is particularly relevant. CIMMYT and partners have already demonstrated success through drought-tolerant (Beyene et al. 2019, 2021) and MLN-resistant (Prasanna et al. 2020; Manigben et al. 2024) hybrids, which have strengthened food security and farmer livelihoods. Building on these successes, integrating high-GCA lines with complementary traits will accelerate the development of next-generation, climate-resilient hybrids for SSA.

The GGE biplot analyses provided clear insights into hybrid adaptation and stability across diverse Kenyan environments. The identification of two distinct mega-environments—one encompassing KYUC (Kirinyaga), Mbeere and Kakamega and another represented by Embu—highlights the need to target breeding and testing strategies for both broad and specific adaptation. Hybrid H1(CKDHL120312/CML312) X CKDHL142124), with its superior yield and wide adaptation across three environments, represents an ideal candidate for advancement as a broadly adapted hybrid for Eastern Africa. In contrast, H18’s (CML395/CML444) X CKDHL143576 specific superiority in Embu suggests it could serve as a location-specific hybrid for regions with similar agro-ecological conditions. The stable and high-yielding hybrids (e.g., H2, H3, H13, H17, H24 and H26) are particularly valuable as parental lines or candidate hybrids for further testing under variable conditions as they combine yield potential with reduced GEI. Conversely, check hybrids (C2, C1, C3 and C6), although stable, showed consistently low yields, underscoring the progress achieved in developing experimental hybrids with greater productivity. Overall, these findings emphasize the utility of GGE biplot analysis in identifying both broadly and specifically adapted hybrids, thereby accelerating genetic gains in stress-prone tropical maize production systems.

The GGE biplot analyses not only revealed patterns of hybrid adaptation but also provided a framework for aligning heterosis utilization with breeding objectives in Eastern Africa (Rezende et al. 2020). The broad adaptation of H1 (CKDHL120312/CML312) X CKDHL142124 across three test environments reflects its superior GCA and capacity to exploit heterosis consistently under diverse conditions, making it a strong candidate for release as a widely adapted hybrid. In contrast, H18’s (CML395/CML444) X CKDHL143576 location-specific superiority in Embu underscores the value of identifying hybrids with high SCA for niche environments, which is critical in stress-prone tropical systems where micro-environmental variation is pronounced. The group of stable and high-yielding hybrids (e.g., H2, H3, H13, H17, H24 and H26) are particularly valuable for advancing into multi-location validation trials and for consideration as future parental pools, as they combine high productivity with reduced GEI. Conversely, the consistently low yields of check hybrids (C2, C1, C3 and C6) highlight the progress made in experimental pipelines, with newer lines surpassing commercial standards in both yield potential and stability. Collectively, these results demonstrate how GGE biplot analyses can guide the strategic deployment of hybrids, either for broad adaptation across heterogeneous environments or for specific adaptation to localized production zones, thereby accelerating the delivery of climate-resilient maize varieties for smallholder farmers in SSA. Importantly, these findings complement the combining ability results by pinpointing hybrids in which strong GCA or SCA aligns with demonstrated adaptation patterns, reinforcing their value for advancement within the breeding pipeline.

### 4.5 | Breeding Implications

This study demonstrates that additive gene action predominates GY and associated agronomic traits, highlighting the importance of exploiting GCA in parental line selection. The strong GCA effects observed in lines such as CKDHL141370, CKDHL142389 and CKDHL143580 confirm their value as key donors for yield, earliness and foliar disease resistance, enabling efficient accumulation of favourable alleles through recurrent selection and line improvement. The identification of high-yielding and stable hybrids (e.g., H1, H2, H3, H13, H17, H24 and H26) reinforces the reliability of GCA-based predictions while also providing promising candidates for advancement in the product pipeline. GGE biplot analyses further revealed two mega-environments, emphasizing the need to balance breeding for broad adaptation (e.g., H1 across KYUC, Mbeere and Kakamega) with targeted deployment of hybrids showing specific adaptation (e.g., H18 in Embu). Together, these results demonstrate that integrating L × T and GGE biplot approaches enables breeders to identify superior parental lines, predict hybrid performance with greater confidence, and strategically deploy hybrids across heterogeneous production environments in SSA. This integrated strategy will accelerate genetic gain and support the delivery of climate-resilient, farmer-preferred maize hybrids.

The early-maturity target product profile has historically been underrepresented in Kenya, largely because it occupies a smaller proportion of the total maize production area. However, increasing climate variability and unpredictable rainfall patterns are driving greater farmer demand for early-maturing varieties that can escape terminal drought and heat stress. Focusing on improving physiological efficiency rather than merely reducing days to flowering is an efficient breeding strategy to develop early maturing varieties. This can be achieved by crossing elite early donors with high-yielding mid-maturity lines while maintaining heterotic structure and selecting within comparable maturity classes (Ertiro et al. 2017). Selection should prioritize traits such as short ASI, rapid grain filling, efficient biomass partitioning and fast grain dry-down. The integration of DH technology, marker-assisted selection for key flowering loci and genomic selection enables rapid recycling and faster genetic gain, while evaluation under managed drought and low-nitrogen environments ensures that early-maturing hybrids retain competitive yield and minimize the typical yield penalty associated with earliness.

## 5 | Conclusion

The L × T analysis confirmed the predominance of additive genetic variance for GY, maturity, PH, EH and foliar disease resistance, underscoring the central role of GCA in predicting hybrid performance. Several elite parental lines, particularly CKDHL141370, CKDHL142389 and CKDHL143580, emerged as multiple-trait donors, combining high yield potential with early maturity and resistance to TLB and GLS, making them valuable resources for future line development and hybrid breeding. Experimental hybrids significantly outperformed commercial checks in yield and disease resistance, with top performers such as H1 showing broad adaptation and H18 demonstrating specific adaptation to Embu. Stable, high-yielding hybrids identified through GGE biplot analyses further provide candidates for multi-location validation and potential release. Collectively, these findings demonstrate that combining ability analyses, complemented by GGE biplot visualization, enables efficient identification of superior parents and hybrids, guiding their deployment across both broad and niche environments. This integrated approach will accelerate the development and dissemination of climate-resilient, high-yielding maize hybrids for smallholder farmers in SSA.

## Supplementary Material

Supplementary Files

Additional supporting information can be found online in the Supporting Information section. **Table S1:** The name of testers, lines and commercial checks used in this study. **Table S2:** BLUEs and BLUPs for 723 testcross hybrids and six commercial checks across eight locations and estimation of variance components. **Table S3:** Estimation of BLUEs and BLUPs for test cross hybrids and commercial checks for grain yield and other traits at each location. **Table S4:** List of best 42 lines used for stability analyses and their code names.

## Figures and Tables

**TABLE 1 |:** The description of the experimental locations used for evaluation of testcross hybrids.

Location	Above sea level (meter)	Longitude	Latitude	Soil type	Rainfall (mm)	Avg temp (°C)
Kakamega	1585	34° 45′ E	0° 16′N	Sandy loam	1996–2214	20.5°C
Kaguru	1450	37° 40′ E	0° 04′S	Sandy loam	600–1200	21°C
Mbeere	1350	37° 50′E	0° 35′S	Clay loam	1450	22°C
Kirinyaga (KYUC)	1297	37° 19′ E	0° 34′S	Clay loam	1100–1250	19°C
Embu	1480	37° 42′ E	0° 49′S	Clay loam	1200–1500	19.5°C
Naivasha	1904	36° 23′ E	0° 41′S	Sandy loam	650	22.6°C
Mabanga	1433	34° 34′N	0° 35′N	Clay loam	356	20.7°C
Shikusa	1570	34° 49′ E	0° 19′N	Sandy loam	1735	20.3°C

**TABLE 2 |:** Estimation of variance components for grain yield and other agronomic traits evaluated in multiple locations in Kenya.

Statistic	GY	MOI	AD	SD	PH	EH	TLB	GLS
*σ* ^2^_G_	0.25*	0.43**	5.74**	4.73**	123.06**	109.59**	0.02*	0.14*
*σ* ^2^_GxE_	0.63**	2.12**	3.28**	4.63**	198.77**	67.80**	0.11**	0.12**
*σ* ^2^_e_	1.96	5.07	6.56	7.58	376.91	156.57	0.20	0.17
H^2^	0.44	0.32	0.81	0.77	0.61	0.79	0.27	0.73
LSD_5%_	1.09	1.55	3.13	3.31	20.37	14.55	0.34	0.63
CV	24.71	10.49	3.29	3.52	8.82	10.93	17.09	18.59

Abbreviations: AD, days to 50% anthesis; CV, coefficient of variation; EH, ear height; GLS, grey leaf spot; GY, grain yield; H^2^, broad sense heritability; LSD, least significant difference; MOI, moisture content; PH, plant height; SD, days to 50% silking; TLB, turcicum leaf blight.

* Significant at *p* < 0.05 probability levels.

** Significant at *p* < 0.01 probability levels.

**TABLE 3 |:** Mean performance of grain yield and other agronomic traits in 25 testcross hybrids plus six checks tested in multiple locations.

Three-way testcross hybrid	GY (t/ha)	MOI (%)	AD (days)	SD (days)	PH (cm)	EH (cm)	TLB (1–9)	GLS (1–9)
(CKDHL120312/CML312) X CKDHL142124								
(CKDHL0089/CML395) X CKDHL141370	7.95	21.35	74.27	76.29	221.37	107.17	1.76	1.63
(CKDHL0089/CKDHL0333) X CKDHL141370	7.94	22.52	74.90	75.07	222.24	109.30	2.02	1.41
(CKDHL0089/CKDHL0333) X CKDHL142389	7.82	24.68	75.23	76.02	226.14	113.63	2.23	1.41
(CML312/CML442) X CKDHL143014	7.67	21.91	78.78	78.33	240.27	119.46	2.71	2.00
(CKDHL0089/CML395) X CKDHL143094	7.67	22.70	80.12	80.63	232.00	128.75	2.26	1.71
(CKDHL0228/CML442) X CKDHL140505	7.66	22.92	80.15	81.56	233.69	125.00	2.77	2.35
(CML395/CML444) X CKDHL142908	7.61	22.42	81.83	81.23	249.33	139.28	2.13	1.81
(CKDHL0228/CML442) X CKDHL143014	7.56	23.12	77.34	76.43	247.88	125.89	2.35	2.05
(CML395/CML444) X CKDHL140230	7.54	22.62	81.53	79.43	218.41	120.86	2.48	1.66
(CKDHL0228/CML442) X CKDHL140502	7.48	22.13	79.35	78.25	246.32	132.64	2.58	2.50
(CKDHL120312/CML312) X CKDHL141047	7.48	19.56	77.27	76.43	252.79	133.68	2.86	3.32
(CKDHL0089/CML395) X CKDHL140229	7.42	21.49	79.52	78.80	225.37	126.59	1.99	1.64
(CKDHL0089/CML395) X CKDHL142908	7.41	21.62	82.38	81.48	235.38	125.71	2.13	1.78
(CML395/CML444) X CKDHL142755	7.41	22.62	82.04	81.68	232.58	130.58	2.14	1.84
(CKDHL120312/CML312) X CKDHL142989	7.41	21.58	76.79	75.70	251.55	138.85	2.85	2.28
(CKDHL0089/CKDHL0333) X CKDHL143580	7.38	22.23	76.92	77.24	220.89	111.72	1.72	1.69
(CML395/CML444) X CKDHL143576	7.37	20.68	75.88	76.86	230.60	125.77	1.97	1.65
(CML395/CML444) X CKDHL141370	7.36	22.70	75.96	77.37	223.88	112.04	1.99	1.84
(CML395/CML444) X CKDHL140944	7.33	21.98	83.43	83.09	241.31	137.36	2.13	1.81
(CKDHL0228/CML442) X CKDHL140549	7.23	22.40	80.07	78.63	206.47	102.94	2.54	2.43
(CKDHL120312/CML312) X CKDHL142981	7.21	19.47	76.02	74.47	250.06	121.34	3.03	2.34
(CKDHL0089/CML395) X CKDHL141115	7.19	24.45	82.66	82.23	235.11	129.51	2.22	1.38
(CKDHL0089/CML395) X CKDHL140234	7.19	22.31	77.37	77.98	237.64	133.23	2.22	1.94
(CML312/CML442) X CKDHL140505	7.17	23.28	80.41	80.56	227.84	119.64	3.03	2.82
Check1 (Duma43)	4.57	19.17	69.50	70.85	223.50	106.38	4.58	3.86
Check2 (DK8031)	4.66	19.00	72.99	74.53	222.35	112.69	4.22	3.44
Check3 (Pioneer3253)	4.97	19.89	77.84	78.29	221.82	114.08	4.37	3.76
Check4 (H517)	5.27	21.50	77.05	79.29	239.17	131.15	4.11	4.03
Check5 (WE1101)	5.53	21.09	75.90	76.58	219.06	109.75	4.07	3.34
Check6 (WH505)	6.34	22.64	81.67	81.39	242.05	128.69	3.85	3.18
Mean	5.67	21.45	77.75	78.13	219.99	114.51	4.26	3.41
Minimum	3.49	18.14	69.50	69.31	176.08	81.65	1.95	1.18
Maximum	8.19	25.18	86.09	85.26	257.66	146.87	6.42	6.41

Abbreviations: AD, days to 50% anthesis; EH, ear height; GLS, grey leaf spot; GY, grain yield; MOI, moisture content; PH, plant height; SD, days to 50% silking; TLB, turcicum leaf blight.

**TABLE 4 |:** Partition of variance components for line by Tester and Baker’s ratio of grain yield and other traits evaluated in multiple locations under optimal conditions.

Trait	GY	MOI	AD	SD	PH	EH	TLB	GLS
Line Variance	0.24**	0.39**	4.55**	2.94**	112.98**	86.17**	0.02*	0.08*
Tester variance	< 0.01	0.04*	1.73*	2.34**	11.46**	8.23**	0.02*	0.10**
Line × tester variance	0.03**	0.01*	0.08**	0.11**	< 0.01	< 0.01	< 0.01	< 0.01
Total genotype variance	0.27	0.40	5.68	4.46	118.94	93.31	0.02	0.17
Additive variance	1.10	1.60	22.73	17.82	475.75	373.23	0.09	0.67
Dominance variance	0.13	0.05	0.33	0.46	0.00	0.00	0.00	0.00
Environmental variance	0.20	0.60	0.81	1.00	45.66	18.26	0.03	0.03
Broad-sense heritability	0.86	0.73	0.97	0.95	0.91	0.95	0.73	0.96
Narrow-sense heritability	0.77	0.71	0.95	0.92	0.91	0.95	0.73	0.95
Baker’s ratio	0.99	0.95	0.84	0.72	0.95	0.95	0.73	0.62

Abbreviations: AD, days to 50% anthesis; EH, ear height; GLS, grey leaf spot; GY, grain yield; MOI, moisture content; PH, plant height; SD, days to 50% silking; TLB, turcicum leaf blight.

* Significant at *p* < 0.05 probability levels.

** Significant at *p* < 0.01 probability levels.

**TABLE 5 |:** GCA estimates of selected best 25 lines and six testers for grain yield and their performance for other agronomic traits.

LINE	GY	MOI	AD	SD	PH	EH	TLB	GLS
DH lines								
CKDHL141370	1.09**	0.20	−2.62**	−1.73*	2.46	−4.87	−0.14	−0.17
CKDHL142389	0.95**	0.80	−2.30**	−1.49	2.72	−1.84	−0.09	−0.34
CKDHL143580	0.81**	0.19	−1.05	−0.81	2.53	−1.51	−0.19	−0.16
CKDHL140502	0.75**	0.43	1.57	1.00	11.54**	9.94*	−0.04	0.18
CKDHL142908	0.74**	0.16	3.14**	2.25**	13.44**	10.33**	−0.09	−0.17
CKDHL142124	0.72**	−0.03	1.45	0.48	11.04	11.99**	−0.02	0.10
CKDHL143576	0.72**	−0.35	−1.99*	−1.70*	−1.26	−0.87	−0.15	−0.33
CKDHL140557	0.71*	−0.15	0.64	0.17	13.38**	14.49**	−0.05	0.06
CKDHL141047	0.68*	−0.05	1.54	1.14	19.08**	17.60**	0.06	0.10
CKDHL144192	0.68*	0.13	−3.21**	−0.92	3.49	−15.08**	−0.02	−0.04
CKDHL140521	0.65*	0.01	1.04	0.40	9.34	11.37**	0.04	0.40*
CKDHL141119	0.65*	0.96*	2.75**	1.88**	3.06	8.74*	0.01	−0.05
CKDHL141526	0.64*	0.00	0.82	0.27	−3.14	3.46	0.04	0.22
CKDHL140229	0.62	0.76	1.17	0.07	0.95	7.02	−0.11	−0.21
CKDHL140944	0.61	0.41	4.10**	3.05**	20.55**	17.77**	−0.13	−0.18
CKDHL142755	0.61	0.38	2.70**	1.67	10.41	10.84**	−0.13	−0.24
CKDHL140230	0.59	0.64	1.63*	0.00	−2.91	1.87	−0.05	−0.14
CKDHL143094	0.56	0.78	2.22**	1.87*	8.86	11.54**	−0.09	−0.24
CKDHL143587	0.55	0.16	−1.57	−0.56	0.59	−4.52	−0.13	−0.21
CKDHL140543	0.54	0.11	1.48	0.47	6.64	9.90*	−0.03	0.10
CKDHL141045	0.53	0.00	−0.57	−0.43	5.75	9.22*	0.01	0.22
CKDHL140234	0.53	0.56	0.01	−0.65	1.45	5.27	−0.08	−0.10
CKDHL143106	0.52	0.12	1.59*	1.01	11.07**	8.50*	0.00	0.06
CKDHL140531	0.51	0.54	−0.26	−0.16	6.43	6.77	−0.01	0.19
CKDHL143014	0.51	−0.01	0.37	−0.22	12.19**	6.02	−0.03	−0.26
CKDHL141087	0.51	0.57	1.15	0.38	2.58	1.47	−0.13	−0.14
Testers								
(CKDHL0089/CKDHL0333)	−0.01	−0.07	0.74*	0.70*	−2.62	−0.94	−0.06	−0.30*
(CKDHL0089/CML395)	0.01	−0.08	0.40*	0.64*	0.80	1.06	−0.12	−0.25*
(CKDHL0228/CML442)	−0.02	0.18	0.17	0.20	−4.19*	−3.70*	0.11	0.10
(CKDHL120312/CML312)	0.00	−0.22	−2.57**	−2.99**	3.26	−0.96	0.11	0.47*
(CML312/CML442)	0.02	0.18	0.26	0.19	1.42	0.03	0.08	0.23*
(CML395/CML444)	0.01	−0.01	1.01*	1.26*	1.33	4.50*	−0.12	−0.25*

Abbreviations: AD, days to 50% anthesis; EH, ear height; EPO, ear position; EPP, ears per plant; GLS, grey leaf spot; GY, grain yield; MOI, moisture content; PH, plant height; SD, silking date; TLB, turcicum leaf blight.

* Significant at *p* < 0.05 probability levels.

** Significant at *p* < 0.01 probability levels.

**TABLE 6 |:** Summary statistics of GCA estimates of lines and SCA estimates of testcross hybrids for grain yield and other agronomic traits evaluated in multiple locations.

Statistics	GY	MOI	AD	SD	PH	EH	TLB	GLS
No. of +ve GCA (%)	118	119	121	124	120	116	131	125
No. of −ve GCA (%)	125	124	122	119	123	127	112	118
Minimum	−0.84	−1.09	−4.79	−4.23	−22.01	−17.08	−0.21	−0.37
Maximum	1.09	1.22	5.01	0.83	21.19	20.92	0.16	0.54
No. of +ve SCA (%)	376	353	252	369	371	371	678	342
No. of −ve SCA (%)	350	373	361	357	355	355	48	384
Minimum	0.21	−0.05	−0.24	−0.35	−0.15	−0.15	−0.01	−0.01
Maximum	0.21	0.05	0.33	0.42	0.12	0.12	0.01	0.01
No. of hybrids	726	726	613	726	726	726	726	726

Abbreviations: AD, days to 50% anthesis; EH, ear height; EPO, ear position; EPP, ears per plant; GLS, grey leaf spot; GY, grain yield; MOI, moisture content; PH, plant height; SD, silking date; TLB, turcicum leaf blight.

## Data Availability

The datasets generated and analysed during this study are available in the Supporting Information.
